# Epithelial‐Mesenchymal Transition, Immune Response, and Extracellular Matrix Remodelling in Oral Cancer

**DOI:** 10.1111/jop.70126

**Published:** 2026-02-12

**Authors:** Nicole Lonni, Camilla Kammer Pereira, Amanda Maciel Costa, Aline Hsiao Bona, Andressa Fernanda Paza Miguel, Elis Ângela Batistella, Daniella Serafin Couto Vieira, Elena Riet Correa Rivero

**Affiliations:** ^1^ Health Sciences Center Federal University of Santa Catarina SC Brazil; ^2^ College of Dentistry, University of Saskatchewan Saskatoon Saskatchewan Canada; ^3^ Department of Pathology, Health Sciences Center Federal University of Santa Catarina SC Brazil

**Keywords:** cancer‐associated fibroblasts, epithelial‐mesenchymal transition, immunohistochemistry, oral squamous cell carcinoma, tumor microenvironment

## Abstract

**Aim:**

This retrospective study investigates the interplay between epithelial‐mesenchymal transition (EMT) and immune response within the tumor microenvironment (TME) of oral squamous cell carcinoma (OSCC).

**Methodology:**

Immunohistochemistry was conducted on OSCC specimens to evaluate the expression of podoplanin (PDP), E‐cadherin (CDH1), vimentin (VIM), fibronectin (FN), tenascin‐C (TNC), and vascular endothelial growth factor (VEGF). Cancer‐associated fibroblasts (CAFs) were identified by alpha‐smooth muscle actin (α‐SMA), and immune cells were quantified using CD66b for neutrophils and CD8 for T lymphocytes.

**Results:**

Higher α‐SMA and moderate‐to‐strong VEGF expression were associated with reduced CDH1 and increased VIM and PDP expression (*p* < 0.05), indicating an EMT‐like phenotype. Cases with elevated PDP—also linked to high α‐SMA—showed increased CD66b^+^ cell density (*p* = 0.072). VEGF expression additionally correlated with greater tumor thickness and depth of invasion (*p* = 0.059). Strong TNC expression was associated with reduced CD8^+^ T‐cell infiltration in the tumor centre and increased CD66b^+^ neutrophils (*p* < 0.046). Strong FN expression was linked to a higher neutrophil‐to‐lymphocyte ratio (*p* = 0.031). Elevated CD66b^+^ cell counts and a higher neutrophil‐to‐lymphocyte ratio were both significantly associated with poorer overall survival (*p* < 0.015).

**Conclusion:**

These exploratory findings suggest that CAFs serve not only as a marker of stromal activation but may also contribute to immunomodulation and invasive tumor phenotype.

## 1 Introduction

Head and neck cancer ranks among the most prevalent malignancies worldwide [1], with oral squamous cell carcinoma (OSCC) accounting for 90% of cases involving the oral cavity [2]. Metastatic disease, responsible for the majority of cancer‐related deaths [3], is largely driven by the epithelial‐mesenchymal transition (EMT), a pivotal process for tumor progression and dissemination [4].

EMT represents the first step in the metastatic cascade, characterized by a phenotypic switch in cancer cells from an epithelial to a mesenchymal state, or even a hybrid state [4]. This transition involves the downregulation of cell–cell adhesion molecules, such as E‐cadherin (CDH1), coupled with the upregulation of mesenchymal markers, like vimentin (VIM), which promote cellular motility and invasion [4]. In addition, EMT alters the tumor cell's antigenic profile, enabling immune evasion by masking or modifying surface peptide presentation, further complicating immune recognition and response [5].

Beyond its direct effects on tumor cells, EMT significantly reshapes the tumor microenvironment (TME) [6]. This complex ecosystem varies between tumor types but universally supports tumor progression [6]. Among the extracellular matrix (ECM) molecules, podoplanin (PDP) [7], tenascin (TNC) [8, 9], and fibronectin (FN) [10] emerge as key players in mediating EMT‐related changes and influencing the immune landscape.

PDP, frequently localized at the invasive tumor front, plays a central role in EMT by promoting the downregulation of CDH1 [11, 12]. PDP expression is not confined to tumor cells; it is also observed in cancer‐associated fibroblasts (CAFs), the most abundant stromal cells within tumors. CAFs possess strong immunomodulatory, angiogenic, metabolic reprogramming, and stemness properties [12, 13]. Moreover, CAFs express alpha‐smooth muscle actin (α‐SMA), which contributes to the recruitment and polarization of tumor‐associated macrophages (TAMs), particularly driving the accumulation of M2 macrophages that foster immunosuppression and dampen T cell activity [13].

FN and TNC, two critical components of the ECM, further contribute to the pro‐tumor TME [10]. FN favours the infiltration of immunosuppressive cell types [8, 10] while TNC disrupts cell adhesion, creating a permissive environment for tumor growth while impairing immune defence mechanisms [9].

To assess the immune response within the TME, this study focused on the analysis of neutrophils and lymphocytes, key players in the immune system. Neutrophils, often implicated in promoting inflammation and facilitating tumor progression [14], were evaluated alongside lymphocytes, which are essential for adaptive immunity and antitumor activity [15]. The balance between these immune cell populations has been shown to reflect the immunological state of the TME and its association with tumour behaviour and prognosis [16, 17].

This study investigated the interaction between EMT markers and the immune response within the TME of OSCC, emphasizing the expression of key molecules such as PDP, CDH1, VIM, FN, and TNC, as well as the presence of cancer‐associated fibroblasts (α‐SMA) and their roles in tumor progression and immune evasion.

## 2 Methodology

### 2.1 Study Design

The study protocol adhered to the principles of the Declaration of Helsinki and was approved by the local Research Ethics Committee (approval number: 17674419.9.0000.0121). This was a convenience sample composed of 31 primary OSCC resection specimens that met the inclusion criteria. Inclusion criteria were primary OSCC, no preoperative chemo‐radiotherapy, and availability of tissue samples. Patients with recurrent or secondary OSCC were excluded. One examiner extracted clinical data from patient files, including age, sex, smoking and drinking habits, tumour site, TNM classification, histopathological grade, follow‐up information, and treatment modality.

Survival outcomes were assessed using overall survival (OS) and disease‐free survival (DFS), defined as the time from treatment initiation to the date of death (OS), recurrence, or metastasis (DFS) or the date of last follow‐up.

### 2.2 Immunohistochemistry

Paraffin‐embedded tissues were sliced into 3‐μm sections. The slides were incubated overnight with the following primary antibodies: α‐SMA (clone 1A4, 1:400, Dako), CD8 (clone C8/144B, ready for use, Dako), CD66 (clone G10F5, 1:500, BD Biosciences), E‐cadherin (CDH1, clone NCH‐38, ready for use, Dako), fibronectin (clone EP5, 1:500, Santa Cruz Biotechnology), pan‐cytokeratin (clone AE1/AE3, 1:50, Santa Cruz Biotechnology), podoplanin (clone D2‐40, ready for use, Dako), tenascin (clone BC‐24, 1:6000, Sigma Aldrich), VEGF (clone C1‐SC 7269, 1:100, Santa Cruz Biotechnology), and vimentin (clone E‐5, 1:800, Santa Cruz Biotechnology). Immunodetection was performed using the EnVision system (Dako Corporation, Carpinteria, CA, USA). Negative controls were conducted by omitting the primary antibody. The slides were scanned using the Axio Scan. Z1 (Carl Zeiss Microscopy, Germany) at 40× magnification, and the images were processed using QuPath open‐source software (v.0.3.2).

### 2.3 EMT Evaluation and ECM Remodelling

The expression of PDP and TNC was evaluated in both the stromal compartment and malignant epithelial tissue. In contrast, VIM and CDH1 were assessed exclusively in the epithelial component. The analysis of FN, TNC, PDP, and α‐SMA expression in the stroma was performed in hot spot areas and categorized into four groups: absent (0%), weak (< 20%), moderate (20%–50%), and strong (> 50%) positivity.

PDP expression in neoplastic cells was classified into three patterns: Pattern 1, membranous expression restricted to the periphery of tumor islands; Pattern 2, membranous and/or cytoplasmic expression extending beyond the periphery of tumor islands; and Pattern 3, weak and diffuse expression in disorganized epithelial tissue. TNC expression in malignant cells, at the invasive front, was classified as positive or negative based on its presence in the nucleus and/or cytoplasm.

**FIGURE 1:**
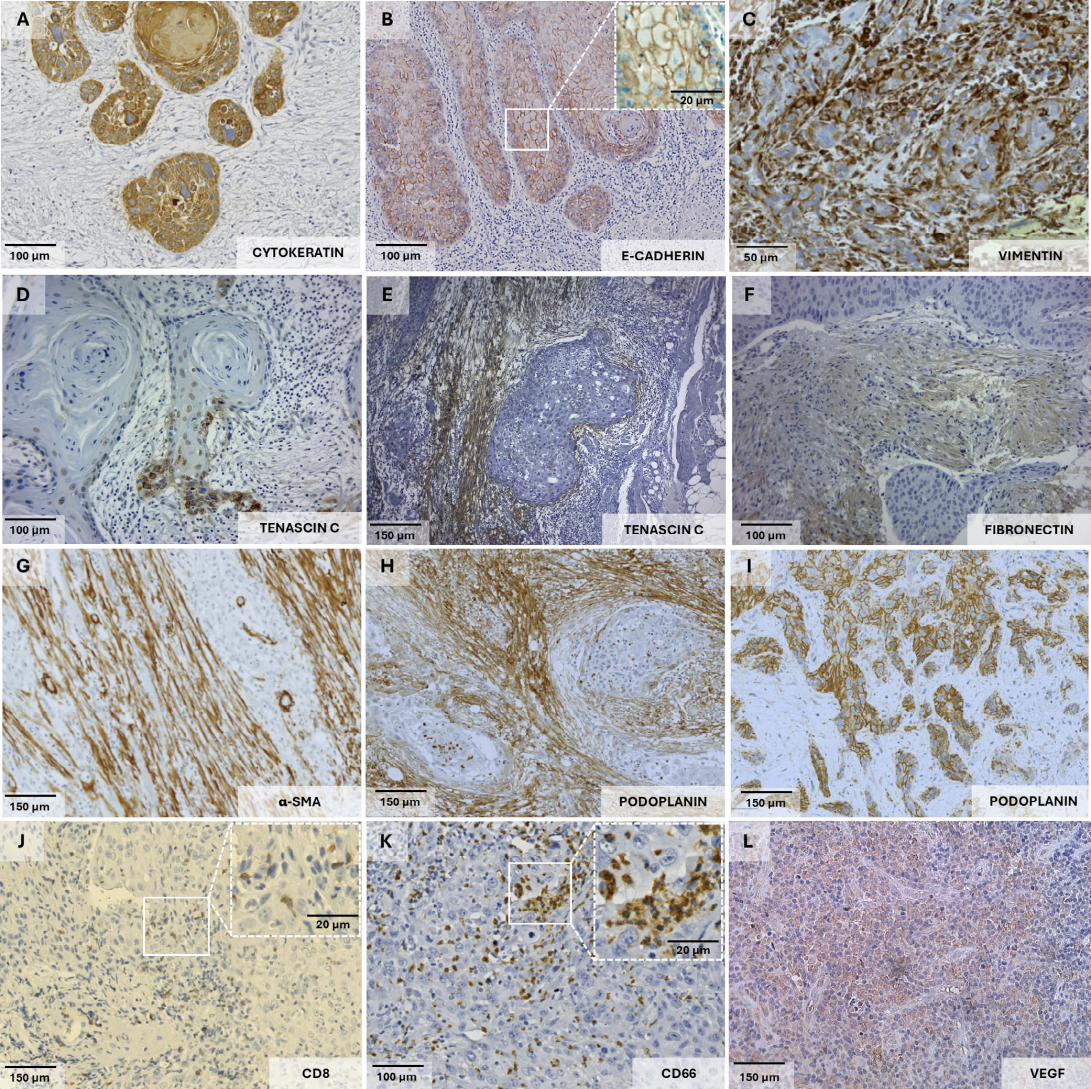
Immunopositive staining in oral squamous cell carcinoma tissue samples demonstrating expression of key markers. (A) Cytokeratin highlights epithelial tumor islands; (B) E‐cadherin; (C) vimentin; (D) tenascin‐C (tumor); (E) tenascin‐C (stroma); (F) fibronectin; (G) alpha‐smooth muscle Actin; (H) podoplanin (stroma); (I) podoplanin (tumor); (J) CD8‐positive T lymphocytes; (K) CD66‐positive neutrophils; (L) vascular endothelial growth factor.

VIM and CDH1 tumoral expression were analyzed in hotspot regions and classified both qualitatively and quantitatively into three categories based on the percentage of positive cells: weak (< 20%), moderate (20%–50%), and strong (> 50%). VIM positivity was defined as cytoplasmic immunoreactivity in epithelial cells, while CDH1 positivity was characterized by strong and continuous membranous staining. TNC was considered positive when nuclear/cytoplasmic staining was observed in tumoral cells. The positivity for all evaluated markers is presented in Figure 1A–I.

### 2.4 Immune Environment and VEGF Evaluation

CD66+ (neutrophil marker) and CD8+ (T lymphocyte marker) counts were performed by selecting 10 representative fields from each case and measuring the number of positive cells in the tumor stroma. The neutrophil/lymphocyte ratio was calculated by dividing the mean number of neutrophils by the number of lymphocytes in each case. The analyses were performed in two specific regions: the invasive front, defined as a 1 mm wide area centred at the tumor borders, and the tumor centre, which encompasses the remaining area. Vascular endothelial growth factor (VEGF) expression was classified as low or absent when the marker was weakly expressed, or moderate to intense when the neoplastic cells were well marked.

Two previously calibrated researchers evaluated the histological sections of each marker individually. When divergent answers or doubts emerged, a third evaluator was called in. The researchers were calibrated through the prior analysis of 30% of the slides. The positivity for the referenced markers is presented in Figure 1J–L.

### 2.5 Tumor Thickness and Depth of Invasion

In samples stained with pan‐cytokeratin, the depth of invasion (DOI) and tumor thickness (TT) were evaluated as previously described [18].

### 2.6 Statistical Analyses

Statistical analyses were performed to explore associations between clinicopathological variables and the markers evaluated. Categorical comparisons were assessed using the *χ* ^2^ test or Fisher's exact test when expected cell counts were low, as these tests are appropriate for evaluating proportional differences between groups. Effect size for these analyses was quantified using Cramér's V (V). Continuous or ordinal non‐parametric data were analysed using the Mann–Whitney *U* test (two groups) or the Kruskal–Wallis H test (three or more groups), with Dunn's test for post hoc pairwise comparisons, given that most variables did not follow a normal distribution. Effect size for Mann–Whitney and Dunn pairwise tests was expressed as Cohen's r (r). Inter‐ and intra‐rater reliability for immunohistochemical scoring were assessed using Cohen's kappa coefficient to quantify agreement beyond chance. Survival probabilities were estimated using Kaplan–Meier curves, and differences between groups were tested with the log‐rank test, which is suitable for comparing time‐to‐event outcomes. CD66+ and CD8+ cell densities were stratified into quartiles to allow evaluation of dose–response patterns in survival analyses. To contextualize the robustness of the findings, post hoc achieved‐power analyses were performed using G*Power. All analyses were conducted using SPSS Statistics 25 (version 17.0, SPSS Inc., Chicago, IL, USA). A two‐sided P value < 0.05 was considered statistically significant.

## 3 Results

Patient demographics and clinicopathological characteristics are detailed in Table 1. Strong TNC expression was the most frequently observed pattern (*n* = 14), while absent/weak expression was the least common (*n* = 3). Cases with strong stromal TNC expression demonstrated a significantly lower density of CD8+ cells in the tumor centre compared to cases with low expression (Median: 279.15 vs. 496.02; *p* < 0.046; strong ES; *r* = 0.51). Additionally, the total CD66+ cell count across the tumor microenvironment was highest in cases with strong stromal TNC expression compared to those with absent expression (Median: 38.03 vs. 6.76; *p* = 0.022; strong ES; *r* = 0.54). Regarding TNC expression in neoplastic cells, only eight exhibited positivity. However, no statistically significant association was observed between the variables of interest.

**TABLE 1:** Clinicopathological parameters of the study cohort.

Independent variables	Age	N	%
Age	40–59	18	58.1
	≥ 60	13	41.9
Sex	Male	24	77.4
	Female	7	22.6
Smoking	No	4	12.9
	Yes	27	87.1
Alcohol	No	6	20.7
	Yes	23	79.3
Site	Tongue	21	67.7
	Mouth floor	10	32.3
Tumour size	T1/T2	22	73.3
	T3/T4	8	26.7
Lymph node	No	20	80.0
Involvement	Yes	5	20.0
TNM stage	TNM I/II	16	64.0
	TNM III/IV	9	36.0
Histopathological	Well	9	29.0
Grading	Moderate	19	61.3
	Poorly	3	9.7
Recurrence	No	19	63.3
	Yes	11	36.7
Death	No	16	55.2
	Yes	13	44.8
Metastasis	No	25	89.3
	Yes	3	10.7

*Note:* % of valid cases.

**FIGURE 2:**
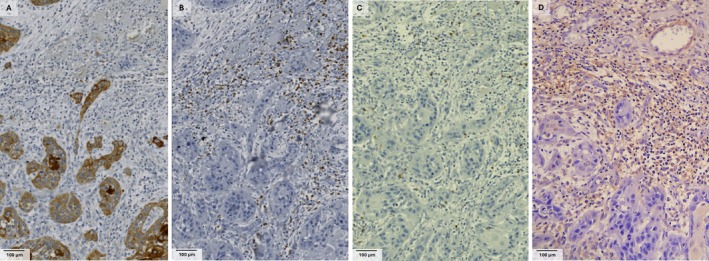
Representative immunohistochemical staining of the same tumor region for Cytokeratin, CD66, and CD8 markers. (A) Cytokeratin staining highlights tumor cells at the invasive front. (B) CD66+ staining reveals high neutrophil concentrations at the invasive front. (C) CD8+ staining shows a low T lymphocyte count. (D) Strong Fibronectin staining.

Stromal FN expression was absent in 15 cases. Notably, the single case exhibiting strong FN expression had a significantly higher neutrophil‐to‐lymphocyte ratio at the tumor invasive front (Figure 2) compared to cases with absent or weak expression (*p* = 0.031; strong ES; *V* = 0.549).

**FIGURE 3:**
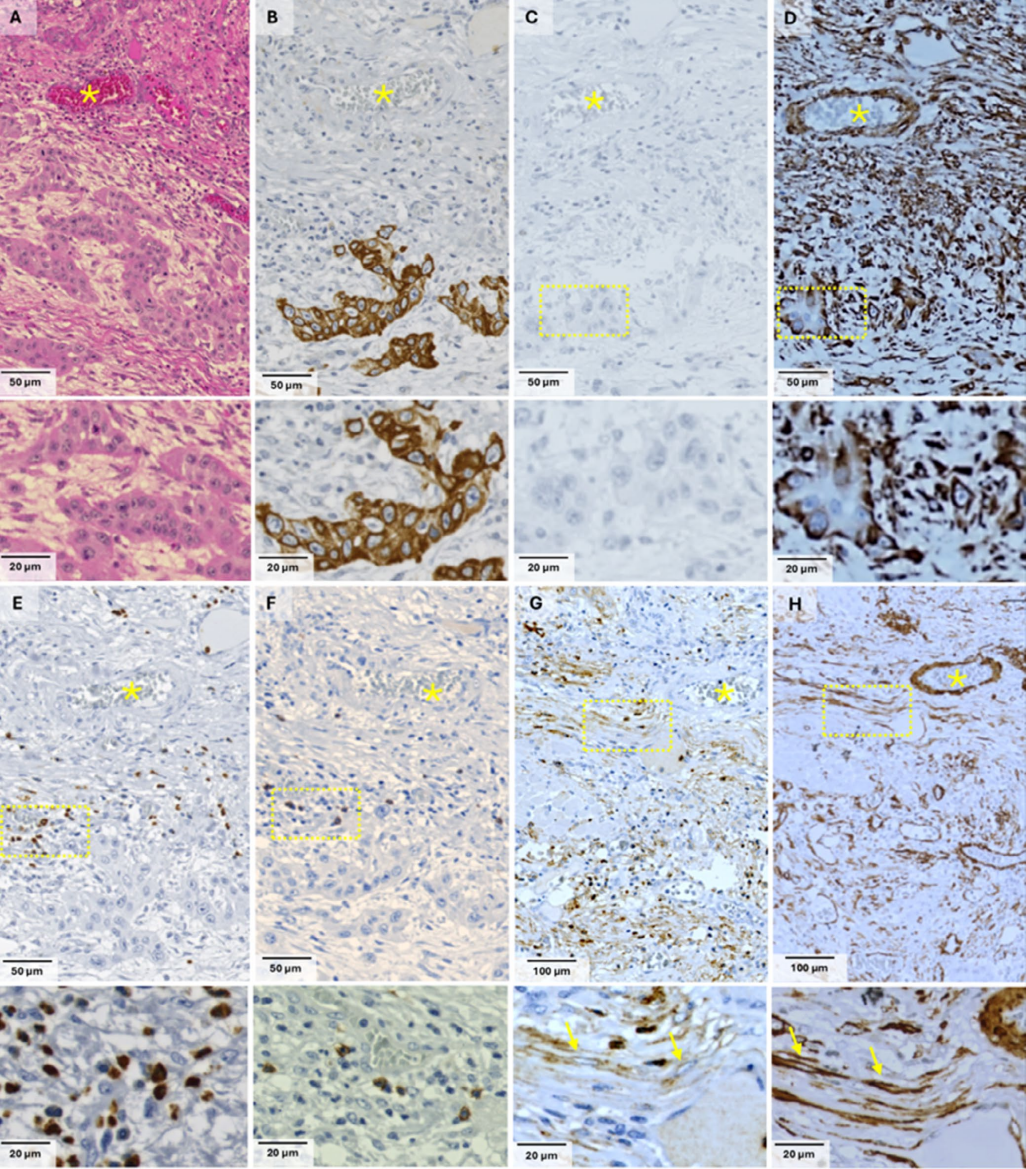
Representative staining of the same tumor region for (A) HE, (B) Cytokeratin, (C) E‐cadherin, (D) Vimentin, (E) CD66, (F) CD8, (G) alpha‐smooth muscle Actin and (H) podoplanin. *A vessel is used as a reference for spatial orientation. The dashed rectangle indicates the region shown at higher magnification. Arrows point to myofibroblasts.

α‐SMA expression showed significant associations with CDH1, VIM, and PDP expression (*p* < 0.05). Higher α‐SMA expression correlated with reduced CDH1 levels (*p* = 0.02; strong ES; *V* = 0.618) and elevated expression of VIM (*p* = 0.05; strong ES; *V* = 0.612) and PDP (*p* = 0.032; low ES; *V* = 0.381) (Figure 3). PDP expression in the stromal region was assessed in 22 cases. Although cases with weak PDP expression displayed a lower number of CD66+ cells compared to those with moderate or strong expression, this difference did not achieve statistical significance (*p* = 0.072). These findings point to a potential α‐SMA–PDP–CD66+ axis, in which a more activated stromal profile (α‐SMA‐high) aligns with increased PDP expression and a trend toward higher neutrophil infiltration.

Tumors with moderate to strong VEGF expression lacked CDH1 expression (*p* = 0.001; moderate ES; *r* = 0.49), while these cases exhibited a higher percentage of VIM‐positive cells (*p* = 0.05; moderate ES; *r* = 0.36). Furthermore, tumors with moderate to strong VEGF expression were characterized by increased thickness and deeper invasion compared to those with absent or weak VEGF expression, though this difference approached but did not reach statistical significance (*p* = 0.059).

**FIGURE 4:**
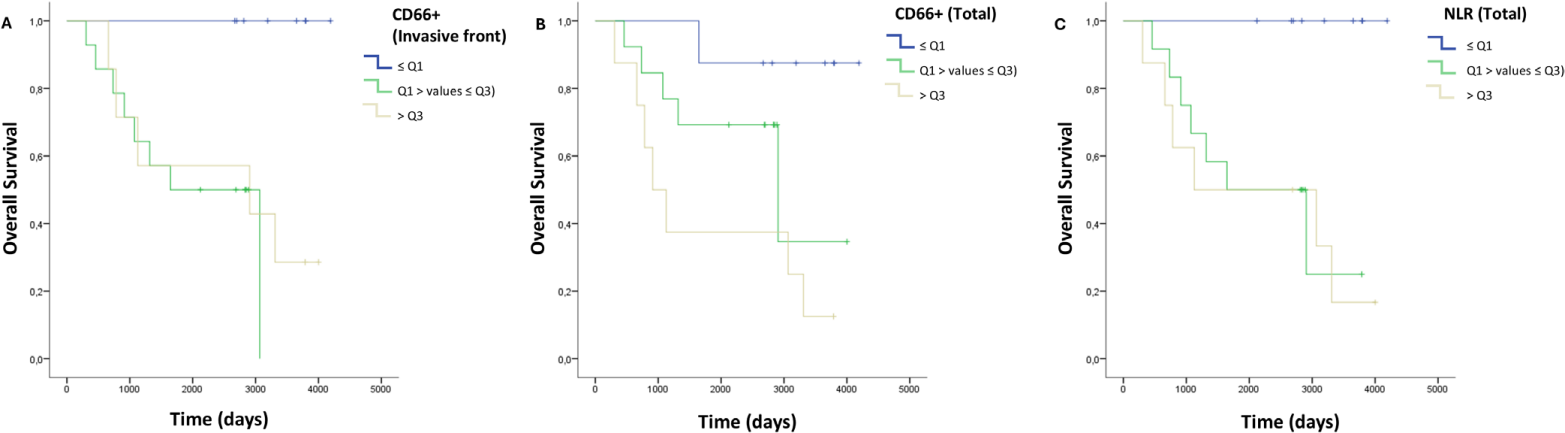
Kaplan–Meier overall survival curves based on immune cell densities. (A) Overall survival stratified by CD66+ cell density in the invasive front, with quartile thresholds at 10.46 (25th percentile), 34.89 (median), and 119.49 (75th percentile). (B) Overall survival stratified by total CD66+ cell density, with quartile thresholds at 6.76 (25th percentile), 23.84 (median), and 72.04 (75th percentile). (C) Overall survival stratified by the neutrophil‐to‐lymphocyte ratio (NLR), with quartile thresholds at 0.05 (25th percentile), 0.12 (median), and 0.56 (75th percentile). Survival probabilities were analyzed using the log‐rank test. Censored data points are indicated by crosses.

Cases with a higher number of CD66+ cells, both at the invasive front and total count, along with an elevated total neutrophil‐to‐lymphocyte ratio, were associated with poorer OS (*p* < 0.015; Figure 4A–C). No significant associations were observed between other variables and patient survival. The summary of immunohistochemical marker expression in OSCC samples is presented in Table 2.

**TABLE 2:** Summary of immunohistochemical marker expression in OSCC samples.

Marker	Expression	*N*	%
*Epithelial expression*			
Podoplanin	Pattern 1	11	42
	Pattern 2	5	19
	Pattern 3	10	38
E‐cadherin	Weak	25	81
	Moderate	3	10
	Strong	3	10
Vimentin	Weak	17	55
	Moderate	4	13
	Strong	10	32
Tenascin	Positive	8	38
	Negative	18	62
VEGF	Absent/Weak	6	26
	Moderate/Strong	17	74
*Stromal expression*			
α‐Smooth‐Actin	Absent/Weak	10	45
	Moderate	1	5
	Strong	11	50
Podoplanin	Absent/Weak	2	9
	Moderate	9	41
	Strong	11	50
Tenascin	Absent	1	12
	Weak	2	19
	Moderate	7	27
	Strong	14	42
Fibronectin	Absent	15	63
	Weak	6	25
	Moderate	2	8
	Strong	1	4

*Note:* % of valid cases.

## 4 Discussion

Disseminated tumor cells must evade immune surveillance to establish and progress. TNC plays a pivotal role in modulating both innate and adaptive immune responses [19]. Strong TNC expression emerged as the predominant pattern in our sample, consistent with previous reports highlighting its persistent expression as a hallmark of various cancers. Notably, we observed that strong TNC expression was associated with a significantly lower density of CD8+ T cells in the tumor centre. This aligns with existing evidence suggesting that a TNC‐rich microenvironment impairs CD8+ T cell function by restricting their mobility, effectively sequestering them within the tumor stroma [8, 20]. This entrapment limits their infiltration into the tumor parenchyma, where they would otherwise exert cytotoxic effects against malignant cells, thereby contributing to immune evasion and tumor progression [8].

Upregulated TNC expression was also associated with an increased total neutrophil count in our samples. Since neutrophils do not biosynthesize TNC, this suggests that its impact on neutrophil behaviour is mediated by exogenous sources [19, 21]. Previous studies have demonstrated that TNC upregulates MMP‐9 synthesis in a TLR4‐dependent manner across various cell types, implicating a potential mechanism through which TNC enhances neutrophil infiltration and activity by increasing MMP‐9 levels [22]. In the tumor microenvironment, elevated TNC expression may foster an immunosuppressive milieu by modulating neutrophil function, potentially promoting an N2‐like pro‐tumoral phenotype, which has been shown to support tumor progression, including in OSCC models [20].

Although TNC is a key regulator of immune responses, its incorporation into the ECM relies on its interaction with FN, a protein frequently overexpressed in various cancers, including OSCC [10]. FN binds to integrins on neutrophils, promoting the formation of neutrophil extracellular traps (NETs) [23]. Notably, the case with the strongest FN expression reported in our findings exhibited a significantly higher neutrophil‐to‐lymphocyte ratio. While originally a host defence mechanism, NETs create a pro‐tumorigenic scaffold that supports tumor progression and metastasis by increasing ECM stiffness and reinforcing FN and TNC deposition in a self‐perpetuating feedback loop [24]. This aligns with our findings that patients with higher CD66+ cell counts, along with an elevated neutrophil‐to‐lymphocyte ratio, exhibited poorer overall survival, consistent with previous studies [16, 17].

Our analysis also revealed strong PDP expression correlated with elevated α‐SMA levels, indicating myofibroblast activation, a finding consistent with previous reports [13, 25]. CAFs play a pivotal and multifaceted role in cancer progression and metastasis by actively shaping the TME [12]. They enhance cancer cell stemness and metastatic potential through paracrine signalling with cancer stem cells, promote angiogenesis by releasing pro‐angiogenic factors, and remodel the extracellular matrix to support vascular growth [11]. Additionally, CAFs mediate immunosuppression by secreting cytokines, which modulate immune responses and facilitate tumor immune evasion [12]. Metabolically, they contribute to the Reverse Warburg Effect, in which cancer cells reprogram neighbouring fibroblasts to undergo aerobic glycolysis and supply lactate/pyruvate that fuels mitochondrial ATP production in tumor cells [12]. Collectively, these functions reinforce CAFs as central players in tumor development and promising targets for therapeutic strategies [12].

CAFs also upregulate PDP in OSCC cells via TGF‐β1/Smad signalling [25]. Mechanistically, PDP facilitates actin cytoskeleton reorganization, an essential process driving tumor cell migration, invasion, and stemness [11]. This interaction plays a pivotal role in PDP‐mediated EMT, tumor progression, lymphangiogenesis, and immune modulation. Furthermore, PDP overexpression has been linked to disrupted adherens junctions, characterized by the loss of CDH1 and a concomitant increase in VIM expression, reinforcing its role in EMT and supporting our findings [11, 25].

PDP expression in CAFs appears to influence neutrophil dynamics, as cases with moderate or strong stromal PDP expression showed higher median CD66+ cell counts. However, this association was not statistically significant and should be interpreted cautiously; the observed trend requires validation in larger cohorts. Recent studies have shown that PDP binding affinity with CD177 (a neutrophil receptor) may be particularly relevant at the CAF‐neutrophil interface, enhancing binding avidity and cellular responses [26]. Additionally, a highly contractile environment acts as a barrier preventing T cell infiltration in the tumor microenvironment.

Building on the pro‐tumorigenic effects of ECM remodelling and immune modulation, VEGF emerges as a key driver of tumor progression through its role in angiogenesis [27]. As a master regulator, VEGF is secreted by epithelial, mesenchymal, and tumor cells, activating endothelial receptors to stimulate vascular remodelling [27]. Cancer progression requires not only cellular transformation but also a supportive vascular network to meet the metabolic demands of tumor expansion. Our findings showed a suggestive trend in which cases with strong VEGF expression had higher TT and DOI, supporting the role of VEGF‐driven angiogenesis in promoting tumor growth and invasion. While the pattern aligns with the recognized role of VEGF‐driven angiogenesis in fostering tumor growth and invasion, confirmation in larger cohorts is required.

Expanding on VEGF's role in tumor progression, its influence extends beyond angiogenesis to actively promote EMT in tumor cells [27]. Given the inherently epithelial nature of OSCC, characterized by strong cell–cell adhesion, invasion and metastasis, it requires the downregulation of adhesion molecules, such as CDH1, while promoting morphological changes and increased motility through VIM upregulation [27, 28]. This mechanistic shift aligns with our findings, as we observed that strong VEGF expression was associated with reduced CDH1 levels, increased VIM expression, and enhanced invasive potential, ultimately contributing to greater DOI and TT. It is crucial to emphasize that phenotypic changes in tumor cells also alter antigen expression, directly impacting their recognition by immune cells [5]. This dynamic modulation may influence immune evasion mechanisms, further shaping the tumor microenvironment and disease progression [5, 29].

Because of the small sample size and the limited number of outcome events, the study had low statistical power, ranging from approximately 9%–72%, depending on the analysis, particularly for detecting effects of small or moderate magnitude. Thus, some non‐significant results may reflect insufficient power rather than the absence of true associations. Even so, several outcomes showed large effect sizes, supporting the internal consistency of the observed patterns. While the limited sample restricts external generalizability, the study still offers meaningful insights into the complexity of the tumor microenvironment and identifies trends that warrant confirmation in larger, adequately powered cohorts.

In conclusion, our findings support the central role of stromal activation, as marked by elevated α‐SMA expression, in shaping the tumor microenvironment and promoting EMT. High α‐SMA expression was significantly associated with reduced CDH1 and increased VIM and PDP expression, reflecting a shift toward a mesenchymal and potentially more invasive tumor phenotype. The overlap between α‐SMA‐associated EMT features and VEGF‐driven tumor characteristics suggests a coordinated stromal‐epithelial signalling axis that may contribute to tumor progression. These changes were accompanied by trends toward increased CD66+ immune cell infiltration, further implicating activated stroma in modulating immune responses. Collectively, our results position α‐SMA as a key indicator of stromal remodelling and a potential therapeutic target within the tumor microenvironment.

## Funding

This work was supported by Coordenação de Aperfeiçoamento de Pessoal de Nível Superior, 001 and Conselho Nacional de Desenvolvimento Científico e Tecnológico (305891/2024‐3, 403444/2023‐3).

## Ethics Statement

This study has obtained the institutional ethical review board (approval number 17674419.9.0000.0121) and the need for informed consent was waived.

## Conflicts of Interest

The authors declare no conflicts of interest.

## Data Availability

The data that support the findings of this study are available on request from the corresponding author. The data are not publicly available due to privacy or ethical restrictions.
